# Cigarette smoke extract induces the senescence of endothelial progenitor cells by upregulating p300

**DOI:** 10.18332/tid/170581

**Published:** 2023-10-03

**Authors:** Guibin Liang, Zhihui He, Huaihuai Peng, Menghao Zeng, Xuefeng Zhang

**Affiliations:** 1 Department of Critical Care Medicine, the Third Xiangya Hospital, Central South University, Changsha, China; 2 Department of Critical Care Medicine, the Second Xiangya Hospital, Central South University, Changsha, China

**Keywords:** p300, cigarette smoke extract, endothelial progenitor cells, senescence

## Abstract

**INTRODUCTION:**

Endothelial progenitor cells (EPCs) are the main source of endothelial cells. The senescence of EPCs is involved in the pathogenesis of chronic obstructive pulmonary disease (COPD). Cigarette smoke extract (CSE) can directly induce the dysfunction and increased expression of senescence-related markers in EPCs cultured in vitro. Histone acetyltransferase p300 is a transcriptional activator, and its changes can lead to cell senescence. The present study investigated whether CSE can induce the senescence of EPCs by upregulating p300.

**METHODS:**

EPCs were isolated from bone marrow of C57BL/6J mice by density gradient centrifugation. The p300 inhibitor C646 and agonist CTPB were used to interfere with EPCs, cell cycle and apoptosis were detected by flow cytometry, the proportion of senile cells was counted by β-galactosidase staining, the protein expression of p300, H4K12, Cyclin D1, TERT and Ki67 were detected by western blot.

**RESULTS:**

Compared with the control group, the cell cycle of CSE group and CTPB group were blocked, the apoptosis rate and early apoptosis rate were increased, the proportion of senile cells counted by β-galactosidase staining was increased, the expression of p300 and H4K12 protein were increased, the expression of Cyclin D1, TERT and Ki67 protein were decreased. C646 could partly alleviate the damages caused by CSE.

**CONCLUSIONS:**

CSE may promote the apoptosis and senescence of EPCs by upregulating the expression of p300 and H4K12 protein, thus preventing the transition of EPCs from G1 phase to S phase, affecting telomerase synthesis, and reducing EPCs proliferation.

## INTRODUCTION

COPD is a chronic respiratory disease, mainly caused by smoking. It seriously harms human health, increases the mortality of patients, and brings heavy economic and social burden to patients and the country. To date, it is generally believed that smoking is one of the key factors in the development of COPD, but its pathogenesis has not been fully clarified. More and more studies have proved that COPD is considered as a disease of premature aging of the lungs^1^. EPCs are derived from mesodermal angioblasts and can differentiate into endothelial cells. EPCs have the biological characteristics of secreting vasoactive substances, proliferation, homing, and migration^2^. EPCs play a key role in postnatal angiogenesis, re-endothelialization, tissue regeneration and repair^3^. Related studies have confirmed that the senescence of EPCs is involved in COPD^4,5^. CSE is a liquid made by passing cigarette smoke through the culture medium, which contains almost all compounds inhaled during smoking. CSE can directly induce the dysfunction of EPCs and increase the expression of aging-related markers *in vitro* ^4,5^. Therefore, it is of great significance to explore the senescence pathway of EPCs caused by CSE.

The acetylation of histones is widespread in eukaryotic cells, and most lysine residues on histones H3 and H4 are continuously acetylated during the whole mitotic period of mammalian somatic cells. The acetylation level of core histone will affect the connection between DNA and core histone, and then affect the transcriptional activity of genes. In lung tissues of mice exposed to cigarette smoke and epithelial cells induced by CSE, the researchers found that histone H4K12 acetylation level was upregulated^6^. H4K12 is an amino acid residue site in histone H4, and other sites include H4K8 and H4K16. H4K12 can loosen the connection between DNA and core histone through acetylation, and enhance the transcriptional activity of genes, thus affecting the expression of many downstream proteins. Histone acetyltransferase p300 is a transcriptional activator with histone acetyltransferase activity, which can upregulate the acetylation level of histone H4K12^7^. The researchers found that the senescence-like changes of endothelial cells can be delayed by knockdown of p300, and the specific mechanism may be through the reduction of the expression of β-galactosidase(β-gal) in endothelial cells^8^. The present study aimed to investigate whether CSE may induce the senescence of EPCs by upregulating p300.

## METHODS

We followed the methods of He et al.^1^ (2016).

### Experimental animals

Animals were from the Shanghai Experimental Animal Center, Chinese Academy of Sciences. EPCs were derived from healthy C57BL/6J male mice aged 4–6 weeks. The study was ratified by Institutional Review Board of Central South University, implemented in keeping with EU Directive 2010/63/EU.

### Extraction of cells

As in a method previously described, EPCs were derived from bone marrow of mouse^9^. First, the bones of mice were isolated after euthanasia, and mononuclear cells were isolated from bone marrow. Then, they were inoculated into culture bottles and cultured in 37^o^C, 95% humidity, and 5% CO2 atmosphere.

For the morphology of EPCs, double staining with fluorescein isothiocyanate-labeled Ulex europaeus agglutinin-1 (FITC-UEA-1, L9006, Sigma, USA) and 1,1’-dioctadecyl-3,3,3’,3-tetramethylindocarbocyanine perchlorate-labeled acetylated low-density lipoprotein (Dil-acLDL, L3484, Eugene, USA) were used to identify the EPCs.

### Configuration method of CSE, C646 and CTPB

According to a previously published method, CSE was prepared^9^. A cigarette is prepared into 20 mL CSE stock solution. According to previously a published method^10^, protocol and MTT assay, C646 (Topscience, T2452, Shanghai, China) was prepared in 12.5 mM solution, and CTPB (Abcam, ab142224, London, UK) was prepared in 25 mM solution.

The EPCs on day 7 of the culture were transplanted to a 6-well plate (1×10^6^ in 2 mL volume per well). Four groups were assigned: control group, CSE group, CSE+C646 group, and CTPB group. In the control group, CSE group and CTPB group, we added 2 mL EGM-2 to each well. In the CSE+C646 group (final concentration of C646 was 12.5 μM), we added 2 mL C646 solution to each well. After being incubated for 24 h, the culture media of the cells were removed and replaced by 2 mL EGM-2 per well in the control group, and by 2 mL 1%CSE solution per well in the CSE group and CSE+C646 group, 2 mL CTPB solution per well in the CTPB group (final concentration of CTPB was 25 μM). After 24 h, the EPCs were used for flow cytometry analysis. The flow chart is shown in Supplementary file Figure S1.

### Flow cytometry analysis

EPCs were collected and re-suspended after different treatments, fixed with 70% ethanol, stored at 4^o^C overnight, and then suspended at 50 μL phosphate buffer solution containing 10 μg/mL RNA enzyme A and 150 μL proridine iodide, and flow cytometry (BD Biosciences, San Jose, USA) was used to detect the cell cycle. EPCs apoptosis was measured with FITC Annexin V and propidium iodide (PI) double staining kit (Invitrogen, 556547, Carlsbad, USA). EPCs were collected and re-suspended after different treatments, then 5 μL PI and 5 μL Annexin V incubation. After dark incubation at room temperature for 20 minutes, EPCs were stained for 15 minutes, and apoptosis was detected by flow cytometry (BD Biosciences, San Jose, USA). Flowjo software (Flowjo, Ashland, USA) were used to analyze the final test data.

### β-Gal staining

Cells were stained with cell senescence detection kit (Millipore, CS0030, Billerica, MA, USA) for SA-gal activity. Then quantitative positive staining was performed with ImageJ.

### Western blot analysis

The protein was electrophorized in gel and transferred to the membrane (Millipore, Billerica, IPVH85R, MA, USA). Incubation of membrane and primary antibody overnight (p300: 1:200, abcam, ab259330, UK; H4K12: 1:200, UK; Cyclin D1: 1:200, abcam, ab134175, UK; Ki67: 1:1000, abcam, ab92742, UK; TERT: 1:1000, abcam, ab32020, UK). Then carefully washed with tris buffer saline and Tween-20 (TBST), and incubated it again with secondary antibody (1:3000,1 h). After washing with TBS-T again, used ECL chemiluminescence substrate to detect the immunoreactive bands (Thermo, 17295, USA).

### Statistical analysis

The data were analyzed by SPSS 16.0 and are presented as mean ± SD. Analysis of differences among groups were performed using analysis of variance (one-way ANOVA). Statistical significance was assumed when p<0.05 or p<0.01.

## RESULTS

### Extraction of EPCs

The cells were suspending in the media (Supplementary file Figure S2A). The cells were oval-shaped and fusiform gradually. There was a tendency to adhere to each other, forming a spherical structure (Supplementary file Figure S2B). Cells were fusiform, polygonal, contacting with each other and trying to form capillary structure after a few more days (Supplementary file Figure S2C). Analysis of double staining showed that the rate of amphoteric cells was 95.34 ± 5.21% (Supplementary file Figure S3).

### Cell cycle

In the Supplementary file Figure S4 are shown the proportions of G1, S and G2 phases of each group of EPCs. Compared with the control group, the proportion of G1/S phase of EPCs cell cycle in CSE group was increased (p<0.05), and the proportion of G1/S phase of EPCs cell cycle in CTPB group was increased (p<0.05). Compared with CSE group, the proportion of G1/S phase of EPCs cell cycle in CSE+C646 group was decreased (p<0.05).

### Cell apoptosis

**Figure 1:**
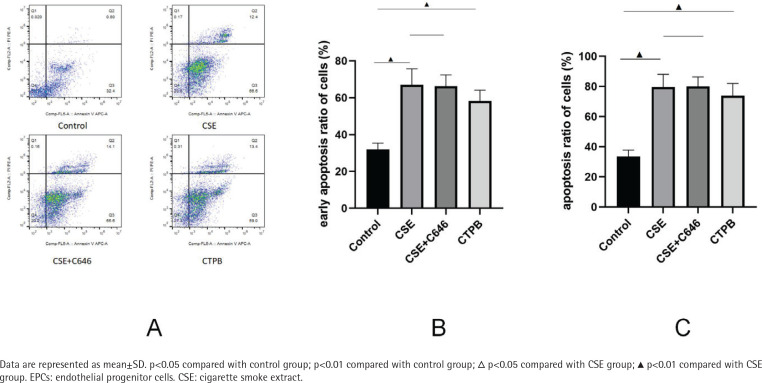
A) The early apoptosis rate and apoptosis rate of EPCs in each group, and B/C) Corresponding quantitative analysis

As shown in Figure 1, compared with the control group, the apoptosis rate of EPCs in CSE group was increased (p<0.01), and the apoptosis rate of EPCs in the CTPB group was increased (p<0.01). Compared with the CSE group, there was no difference in the apoptosis rate of EPCs in the CSE+C646 group (p>0.05). Compared with the control group, the early apoptosis rate of EPCs in the CSE group was increased (p<0.01), and the early apoptosis rate of EPCs in the CTPB group was increased (p<0.01). Compared with the CSE group, there was no difference in the early apoptosis rate of EPCs in the CSE+C646 group (p>0.05).

### β-Gal staining

As shown in Supplementary file Figure S5, compared with the control group, the positive rate of β-galactosidase in EPCs in the CSE group was increased (p<0.01), and the positive rate of β-galactosidase in EPCs in CTPB group was increased (p<0.01). Compared with the CSE group, there was no difference in the positive rate of β-galactosidase in EPCs in the CSE+C646 group (p>0.05).

### Expression levels of p300, H4K12, Cyclin D1, Ki67 and TERT protein

**Figure 2:**
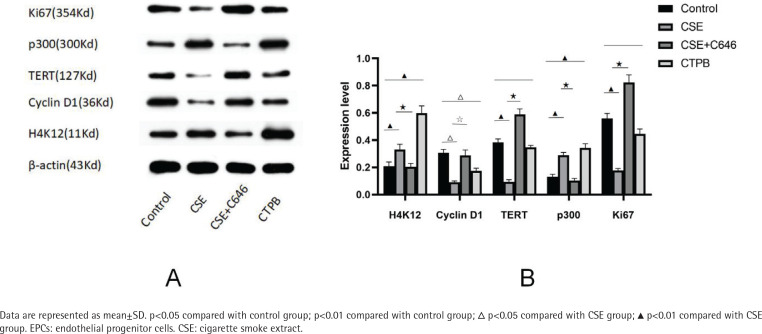
A) The expression levels of Ki67, p300, TERT, Cyclin D1 and H4K12 protein in EPCs, and B) Corresponding quantitative analysis

As shown in Figure 2, compared with the control group, the relative expression level of H4K12 protein in the CSE group was increased (p<0.01), and the relative expression level of H4K12 protein in the CTPB group was increased (p<0.01). Compared with the CSE group, the relative expression level of H4K12 protein in the CSE+C646 group was decreased (p<0.01). Compared with the control group, the relative expression level of Cyclin D1 protein in the CSE group was decreased (p<0.05), and the relative expression of Cyclin D1 protein in the CTPB group was decreased (p<0.05). Compared with the CSE group, the relative expression level of Cyclin D1 protein in the CSE+C646 group was increased (p<0.05). Compared with the control group, the relative expression of TERT protein in CSE group was decreased (p<0.01), and there was no difference in the relative expression of TERT protein in the CTPB group (p>0.05). Compared with the CSE group, the relative expression of TERT protein in the CSE+C646 group was increased (p<0.01). Compared with the control group, the relative expression level of p300 protein in the CSE group was increased (p<0.01), and the relative expression level of p300 protein in the CTPB group was increased (p<0.01). Compared with the CSE group, the relative expression level of p300 protein in the CSE+C646 group was decreased (p<0.01). Compared with the control group, the relative expression level of Ki67 protein in the CSE group was decreased (p<0.01), and there was no difference in the relative expression level of Ki67 protein in the CTPB group (p>0.05). Compared with the CSE group, the relative expression level of Ki67 protein in the CSE+C646 group was increased (p<0.01).

## DISCUSSION

The results of the present study show that G1/S phase transition arrest occurred in the CSE group and the CTPB group, while C646 can relieve the influence of CSE on cell cycle, suggesting that p300 can affect the cell cycle of EPCs. CSE can increase the early apoptosis rate of EPCs. Although there is no difference between the CSE+C646 group and the CSE group in the early apoptosis rate of EPCs, CTPB can increase the early apoptosis rate of EPCs compare with the control group. CSE can increase the positive rate of β-galactosidase in EPCs, and there is no difference between the CSE+C646 group and the CSE group in the positive rate of β-galactosidase in EPCs, and CTPB can increase the positive rate of β-galactosidase in EPCs compared with the control group. The protein expression of CyclinD1, TERT and Ki67 are opposite to p300, and the protein expression of K4K12 is the same as p300. The results firstly confirm that CSE may promote the apoptosis and senescence of EPCs by upregulating the expression of p300 and H4K12 protein, thus preventing the transition of EPCs from G1 phase to S phase, affecting telomerase synthesis, and reducing EPCs proliferation.

Apoptosis refers to the whole process of cell running inside, which leads to the cell stop growing, developing and decomposing, and finally makes the cell die controllably. It is characterized in that substances in cells are not easy to leak into the surrounding environment due to death. Apoptosis plays a key role in the aging process of animal cells including human beings. Some diseases related to aging are caused by the accumulation of toxic and side effects of protein aggregates, which can induce pathological apoptosis^11^. In the regulation of cell cycle, the transition from G1 phase to S phase is the most important regulatory point. If this regulation point is abnormal, the proliferation of cells will become out of control, which can lead to cell senescence. Cell aging is characterized by an exponential decrease in the percentage of cells that can synthesize DNA and increase cell cycle time^12^. Although the initial stage of cell division determines the state of proliferation and division, most adult cells remain in a static state (G0 stage), and cells usually enter a static state after mitosis or before terminal differentiation. Cell cycle transition is driven by cyclin and cyclin-dependent kinases (CDKs)^13^. CyclinD1 promotes the progression of the cell cycle in the G1 phase and is the key protein for G1/S phase transition. The gene CCND1 encode that protein by the Q13 band located on chromosome 11q13. CCND1 gene and its protein CyclinD1 are often changed by different molecular mechanisms, including amplification, chromosome translocation, mutation and activation of CyclinD1 expression pathways, which are essential in the occurrence and development of human cancer and aging. In 2016, Tang et al.^14^ found that the expression of anti-aging gene Klotho increased in patients with liver cancer, which inhibited Wnt/β-catenin signaling pathway, decreased the expression of β-catenin, inhibited the transport of β-catenin from cytoplasm to nucleus, and decreased the expression of two known target genes c-myc and CyclinD1 in Wnt/β-catenin pathway^14^. The research of Lim^15^ showed that TIS21/BTG2/PC3 could inhibit the expression of CyclinD1 in a pRB and p53-dependent manner, resulting in cell arrest in G1/S phase. On the other hand, TIS21/BTG2/PC3 can inhibit the degradation of cyclin A and cyclin B1 in G2/M phase when stimulated by epidermal growth factor (EGF) at high concentration, and directly combine with Cdc2, thus hindering cell mitosis and increasing the aging and death of tumor cells^15^. Ki67 exists in G1, S and G2 phases of the cell cycle, but does not exist in G0 phase of resting or resting cells, so the expression level of Ki67 reflects the proliferation state of cells^16^. The expression of Ki67 has been proved to be related to aging and the loss of the ability of aging cells to proliferate and form colonies^17^. Lawless et al.^18^ found that loss of Ki67 activity combined with high density DNA lesion (CH2AX) (>5 lesions/nucleus) can be used as the gold standard for aging detection. El-Far et al.^19^ found that quercetin can upregulate the expression of Ki67 in mouse organs and delay aging, which is a promising natural protective compound. In 2020, Prajit et al.^20^ found that the expression of Ki67 decreased in the mouse brain aging model induced by D-galactose, and the corresponding aging degree could be slowed down after intervention with chrysin. Telomerase is composed of telomerase RNA (TR), telomerase reverse transcriptase (TERT) and telomerase related proteins-1 (TEP1), which is a ribonucleoprotein complex. Telomerase uses RNA carried by its own TR as a template, synthesizes telomere repeat sequences to the ends of chromosomes under the catalysis of TERT, prolongs or stabilizes telomeres which shorten progressively with cell division, and plays a key role in the occurrence and development of cell aging^21^. TERT is a catalytic protein subunit of telomerase, which maintains the integrity of chromosomes and the stability of the genome^22^. Telomerase is expressed more in germ cell lines and stem cells, but less in most somatic cells. In the latter, telomere loss occurs in every cell division. When the telomere reaches the critical length, cells will go into senescence, and the life span of mice with telomerase deficiency will gradually shorten after continuous hybridization^23^. Tomás-Loba et al.^24^ found that the overexpression of TERT in transgenic mice can improve the adaptability of the epithelial barrier, especially skin and intestinal tract, and lead to the delay of body aging, accompanied by the extension of the median life span, indicating that TERT has anti-aging activity in the mammalian body. Zhang et al.^25^ found that homocysteine accelerated endothelial cell aging through hypomethylation of human telomerase reverse transcriptase DNA. Liu et al.^26^ found that the mouse model of Type II alveolar epithelial cell (AECII) specific TERT deficiency can enhance pulmonary fibrosis by weakening the ability of epithelial regeneration caused by cell aging. The typical biochemical characteristic of aging cells is the enhancement of PH-dependent β-galactosidase expression activity, namely senescence-associated β-galactosidase (SA-gal). The increasing level of SA-gal is proportional to the proportion of aging cells^27^. β-galactosidase staining is one of the most commonly used methods to evaluate cell aging, which can mark aging cells in vivo and in vitro, and is considered as the gold standard for detecting aging^28^.

p300 is an important epigenetic regulator, which plays a key role in cardiac embryogenesis. The lack or abnormal expression of p300 will lead to the deformation and death of the heart and neural tube in early pregnancy. The pharmacological or genetic normalization of p300 activity can prevent or prevent the pathological progress of cardiac aging. The development of safe, non-toxic, p300-targeted small molecule inhibitors is an ideal method to control accelerated death associated with cardiac aging worldwide^29^. Mortuza et al.^30^ found that hyperglycemia can accelerate the aging-like changes of endothelial cells and tissues, leading to structural and functional damage, and p300 knock-down can correct these abnormalities and slow down the aging-like changes. Histone acetyltransferase and Histone deacetylases (HDACs) respectively play a control role in the acetylation process of histone, and add or remove acetyl groups in histone^31^. Histone acetylation occurs at different lysine sites, among which H4K12 can participate in cell aging^32^. The research of Kumar et al.^33^ showed that p300 can increase the acetylation degree of H4K12. Our study found that with the change of p300, H4K12 also changed, indicating that p300 can also affect the acetylation degree of H4K12 in EPCs.

In a previous study, the preparation of p300 overexpression plasmid and silencing plasmid was difficult and the cell transfection efficiency was low, so the p300 inhibitor C646 and agonist CTPB were chosen in the present study. C646, as a competitive inhibitor of p300, can antagonize acetyl coenzyme a, which is the substrate of acetylation of p300 and exerts anti-tumor activity in many cancer cell lines such as prostate cancer and leukemia. In several melanoma and lung cancer cell lines, C646 can slow down cell growth and hinder histone acetylation in cells^34^. Yan et al.^35^ found that C646 was proved to promote the sensitivity to DNA damage drugs, which led to increased apoptosis of melanoma cells after cisplatin combined treatment. N-(4-Chloro3-Trifluoromethyl-phenyl)-2-Ethoxy-6-Pentadecyl-Benzamide (CTPB) is a benzamide which activates p300/CBP HAT activity. CTPB can enhance p300/CBP-dependent transcriptional activation^36^. In the present study, compared with the CSE group, the proportion of EPCs cell cycle G1/S in the CSE+C646 group decreased, indicating that C646 could partially relieve the blocking of EPCs cell cycle by CSE.

Compared with the CSE group, there is no difference in EPCs apoptosis rate and early apoptosis rate, and there is no difference in EPCs β-galactosidase positive rate in the CSE+C646 group. It may be that C646 can partially relieve the influence of CSE on EPCs apoptosis rate, early apoptosis rate and β-galactosidase positive rate, but due to its own toxicity to cells, there is no difference in EPCs apoptosis rate, early apoptosis rate and β-galactosidase positive rate. Compared with the CSE group, the amount of p300 and H4K12 protein in EPCs of the CSE+C646 group decreased, while the amount of CyclinD1, TERT and Ki67 protein increased. C646 could affect the relative expression of p300, H4K12, CyclinD1, TERT and Ki67 protein in EPCs. Compared with the CSE+C646 group, the relative expression of p300, H4K12 and CyclinD1 protein after CTPB intervention increased, while TERT and Ki67 protein had no obvious change, which indicated that CTPB could partly affect the relative expression of p300, H4K12, CyclinD1, TERT and Ki67 protein in EPCs. Compared with the control group, the proportion of EPCs in G1/S phase in the CTPB group is higher, indicating that CTPB may prevent EPCs from entering S phase from G1 phase by increasing the relative expression of p300 protein. Compared with the control group, the apoptosis rate and early apoptosis rate of EPCs in the CTPB group were higher, indicating that CTPB might decrease EPCs proliferation by increasing the relative expression of p300 protein. Compared with the control group, the β-galactosidase positive rate in CTPB group is higher, which may be related to CTPB increasing the relative expression of p300 protein and promoting the aging of EPCs.

## CONCLUSIONS

The present study demonstrated that CSE may promote the apoptosis and senescence of EPCs by upregulating the expression of p300 and H4K12 protein, thus preventing the transition of EPCs from G1 phase to S phase, affecting telomerase synthesis, and reducing EPCs proliferation. The present study may provide a novel understanding in the pathogenesis of emphysema or COPD and systematic impact of cigarette smoking on the human body.

## Data Availability

The data used to support the findings of this study are available from the corresponding author upon request.
