# Supplementary material for: Cigarette smoke extract induces the senescence of endothelial progenitor cells by upregulating p300
Source: Tob Induc Dis. 2023 Oct 3;21:122. doi: 10.18332/tid/170581 (PMC10546488; doi:10.18332/tid/170581)
Supplement: Supplementary file 1 [file TID-21-122-s1.pdf]

Figure S1. Flow chart

EPCs, endothelial progenitor cells; CSE, cigarette smoke extract; WB, western blot.

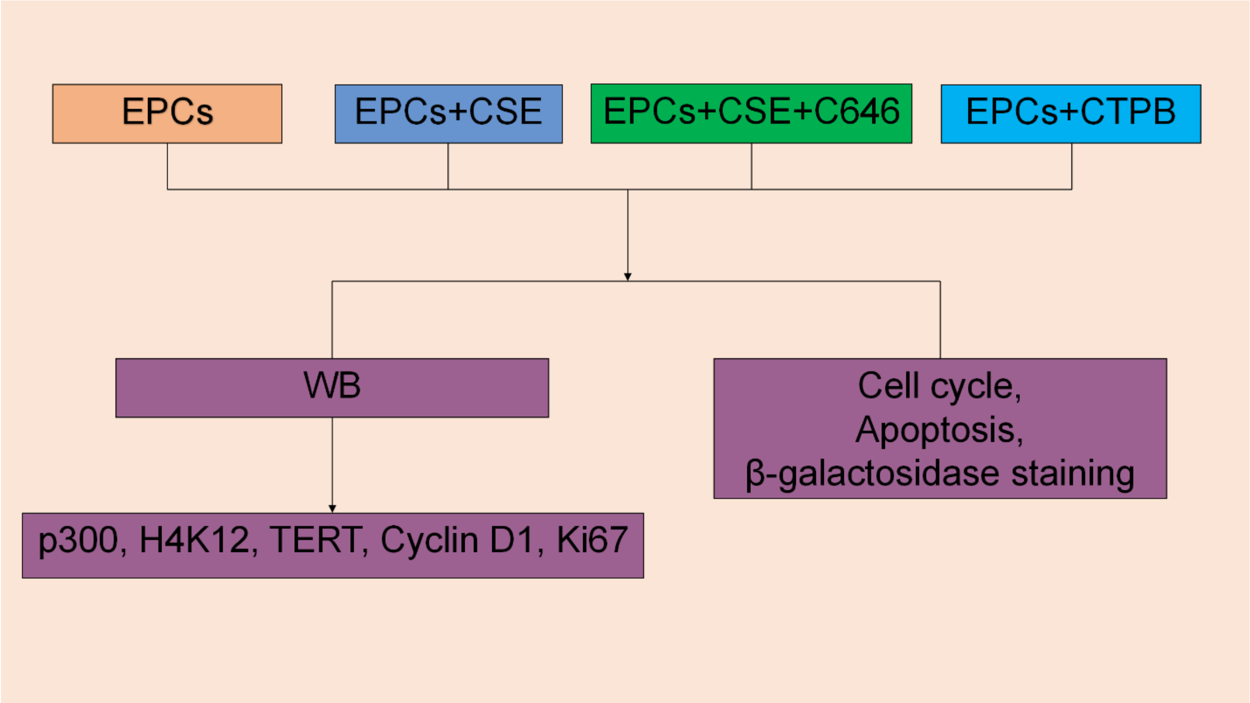

Figure S2. The morphological changes of EPCs sourced from bone marrow during culture.

On day 1 of the culture, the cells were round, similar, and suspended in media (A). On day 4 of the culture, the cells attempted to attach to one another, their sizes increased, and they were oval, spindle or polygonal in shape(B). On day 7 of the culture, the cells followed either fusiform or polygonal patterns and contacted each other in an attempt to form a capillary structure(C). The scale bar represents 50 μm.

EPCs, endothelial progenitor cells.

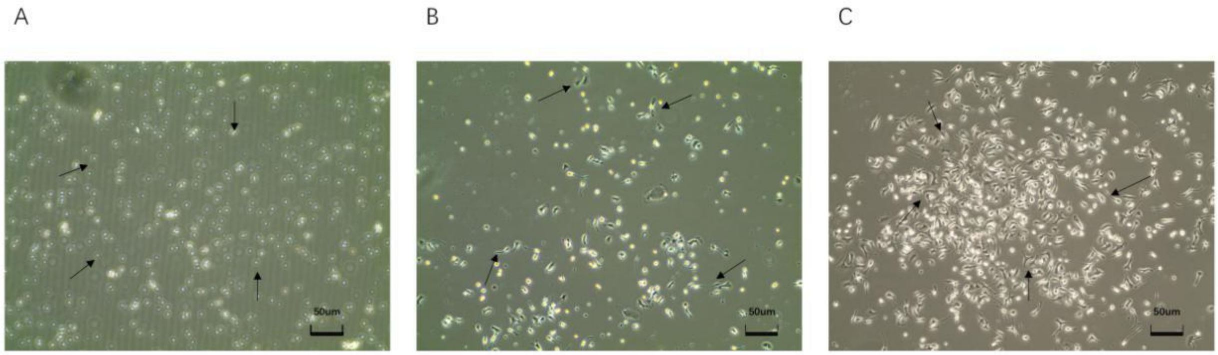

Figure S3. The identification of EPCs by double positive staining with Dil-acLDL and FITC-UEA-1.

On the 7th day of culture, the EPCs were observed with laser scanning confocal microscope (LSCM). When the EPCs absorbed Dil-acLDL, the cytoplasm was red (A), when FITC-UEA-1 was combined, the cytoplasm was green (B), when Dil-acLDL and FITC-UEA-1 were positive stained, the cytoplasm was orange (C), and when DAPI was used for nuclear localization, the cytoplasm was blue (D). Scale bar is 10 μm.

EPCs, endothelial progenitor cells; DAPI, 4',6-diamidino-2-phenylindole. Dil, 1,1'-dioctadecyl-3,3,3',3- tetramethylindocarbocyanine perchlorate; acLDL, acetylated low density lipoprotein; FITC, fluorescein isothiocyanate; UEA-1, ulex europaeus agglutinin-1;

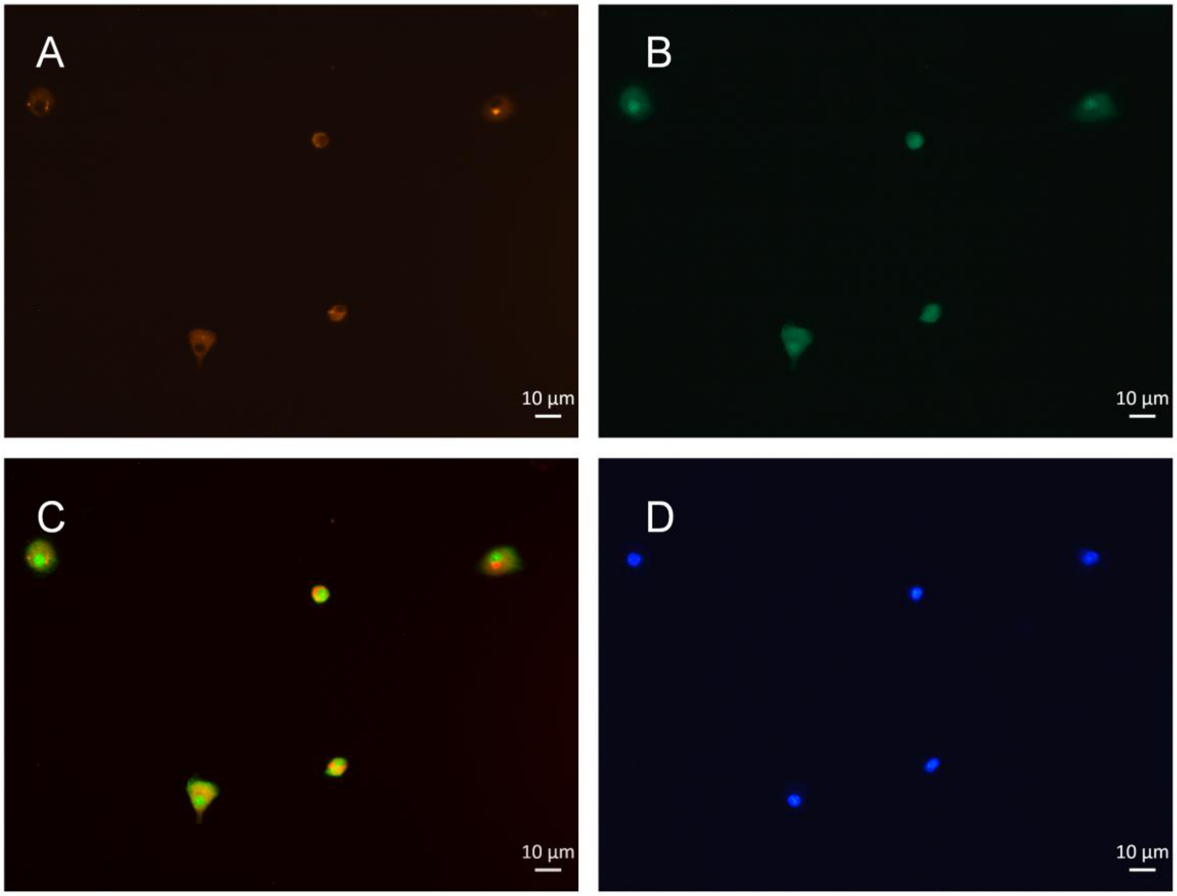

Figure S4. The proportion of G1, S, G2 phase of EPCs in each groups. There were pictures of the proportion of G1, S, G2 phase in EPCs (A), and corresponding quantitative analysis (B). Data are represented as mean±SD. △p<0.05 compared with control group; ▲p<0.01 compared with control group; ☆p<0.05 compared with CSE group; ★p<0.01 compared with CSE group.

EPCs, endothelial progenitor cells; CSE, cigarette smoke extract;

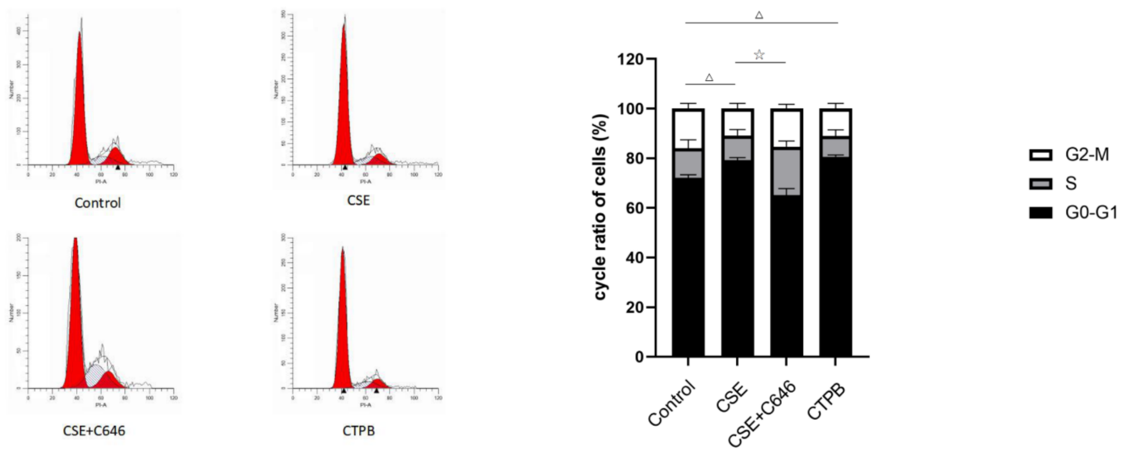

A B

Figure S5. The positive rate of β-galactosidase of EPCs in each groups. There were pictures of the positive rate of β-galactosidase in EPCs (A), and corresponding quantitative analysis (B). Data are represented as mean±SD. △p<0.05 compared with control group; ▲p<0.01 compared with control group; ☆p<0.05 compared with CSE group; ★p<0.01 compared with CSE group.

EPCs, endothelial progenitor cells; CSE, cigarette smoke extract;

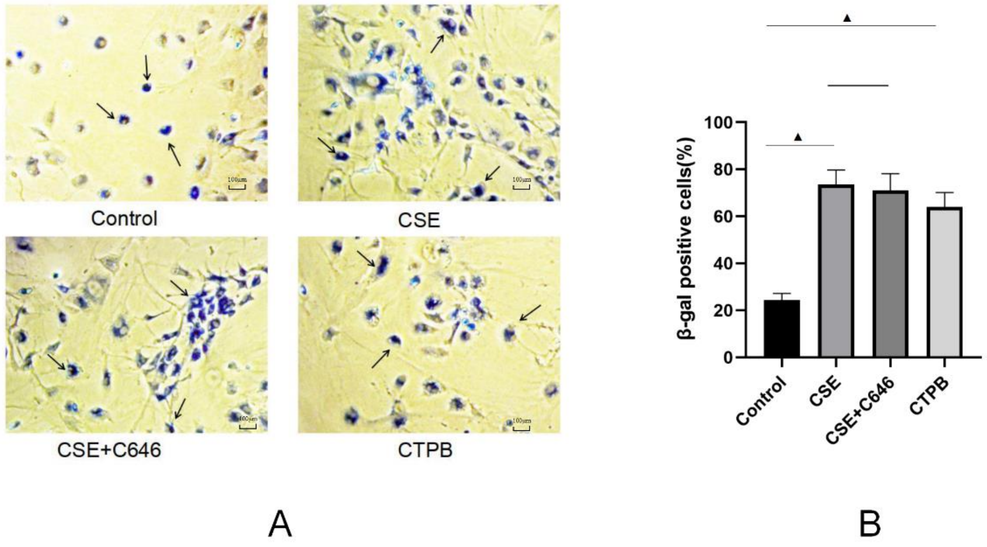

© 2023 Liang G. et al.
